# NLR, A Convenient Early-Warning Biomarker of Fatal Outcome in Patients With Severe Fever With Thrombocytopenia Syndrome

**DOI:** 10.3389/fmicb.2022.907888

**Published:** 2022-06-23

**Authors:** Yuanyuan Wei, Zilong Wang, Luyang Kang, Lingling He, Nan Sheng, Jiangfeng Qin, Shuangshuang Ma, Honghai Xu, Lifen Hu, Guizhou Zou, Yufeng Gao, Jiabin Li

**Affiliations:** ^1^Department of Hospital Infection Prevention and Control, The First Affiliated Hospital of Anhui Medical University, Hefei, China; ^2^Anhui Center for Surveillance of Bacterial Resistance, The First Affiliated Hospital of Anhui Medical University, Hefei, China; ^3^Department of Infectious Diseases, The First Affiliated Hospital of Anhui Medical University, Hefei, China; ^4^Department of Infectious Diseases, The Second Affiliated Hospital of Anhui Medical University, Hefei, China; ^5^Department of Pathology, The First Affiliated Hospital of Anhui Medical University, Hefei, China

**Keywords:** severe fever with thrombocytopenia syndrome, neutrophil-to-lymphocyte ratio, SFTSV viral load, prognosis factors, fatal outcome

## Abstract

**Background:**

Severe fever with thrombocytopenia syndrome (SFTS) is an emerging infectious disease that greatly threatens public health. This study aimed to examine a convenient early-warning biomarker of fatal outcomes in patients with SFTS to reduce mortality.

**Methods:**

A retrospective cohort study was performed, and patients with confirmed SFTS were enrolled in the top two hospitals in Anhui Province, China from 1 May 2016 to 31 October 2019. The clinical symptoms, laboratory indicators, and treatment data of patients with SFTS were evaluated. All patients with SFTS were followed up till 28 days from the start of admission. The laboratory indicators that could be used to predict the fatal outcome were identified.

**Results:**

A total of 228 patients with SFTS were enrolled, 177 patients were enrolled in the survival group, and 51 patients in the death group. The median age of all 228 patients with SFTS was 63 years. Five laboratory indicators (SFTSV viral load, neutrophil to lymphocyte ratio (NLR), aspartate transaminase (AST)/alanine aminotransferase (ALT), ALT, and blood urea nitrogen (BUN)) were identified as the predicting factors of the fatal outcome of patients with SFTS. The area under the receiver operating characteristic (ROC) curve (AUC) of SFTSV viral load was the highest (0.919), then NLR (0.849), followed by AST/ALT (0.758), AST (0.738), and BUN (0.709). The efficacy of SFTVS viral load and NLR in predicting fatal outcomes was significantly higher than AST/ALT, AST, and BUN. The Kaplan–Meier survival curves show that the case fatality rate was significantly increased in patients whose SFTSV viral load was higher than 500,000 or NLR higher than 2.0. Gamma-globulin treatment showed a significant difference between the survival group and the death group, and the duration of gamma-globulin that had been proposed should not be <3 days.

**Conclusion:**

The SFTSV viral load and NLR showed great efficacy in predicting the fatal outcome of patients with SFTS, and NLR is a convenient and efficient early-warning biomarker that helps healthcare workers focus on patients with high risks of fatal outcomes. The efficacy of gamma-globulin provided a new idea for the treatment of SFTS, which needs further analysis in future studies.

## Introduction

Severe fever with thrombocytopenia syndrome (SFTS) is an emerging highly lethal infectious disease caused by SFTS virus (SFTSV), which has been renamed *Dabie bandavirus* that belongs to the genus *Bandavirus* in the family *Phenuiviridae* of the order *Bunyavirales* (Paules et al., 2018; Casel et al., 2021). SFTS has been reported to be mainly transmitted by contact with ticks. Moreover, previous evidence suggested that SFTS could be transmitted from person-to-person (Jung et al., 2019). Since SFTS was first identified in Central China in 2010 and subsequently reported in South Korea, Japan, and Vietnam (Yu et al., 2011; Lin et al., 2020; Li et al., 2021). The growing number and geographic distribution of SFTS cases in recent decades have threatened public global health. Recent updates indicate that the laboratory-confirmed SFTS cases were increased in China to 7,721 by 2018, 1,089 in South Korea by 2019, and 573 in Japan by 2020 (Miao et al., 2021; Zhao et al., 2021). The World Health Organization (WHO) has listed SFTS as one of the top 10 priority infectious diseases due to its high mortality rate, wide geographical distribution, economic burden, and the possibility it could cause a pandemic.

Anhui Province is located in Central China, which is one of the provinces where the first report of SFTS was made. Most endemic regions of the epidemiology of SFTS in China have shown a significant disease burden of SFTS (Bopp et al., 2020). Notably, previous studies had confirmed that SFTS already existed in Anhui province in September and November of 2006 through the detection of SFTSV RNA by real-time PCR (RT-PCR) (Liu et al., 2012). A previous study had indicated that from 2010 to 2017, a total number of 1,506 SFTS cases and 7 human-to-human transmissions have been reported in Anhui Province (Gong et al., 2018). According to previous findings in Anhui Province, the economic burden was significantly high for patients with SFTS and their families, and effective preventive measures are necessary for endemic areas (Gong et al., 2019).

The clinical features of SFTS are not specific, which include fever, headache, myalgia, nausea, vomiting, diarrhea, thrombocytopenia, leukocytopenia, and hemorrhage. Furthermore, when severe, it can present severe symptoms, such as neurological symptoms, bleeding, hemophagocytic syndrome, disseminated intravascular coagulation, multiorgan failure, and death (Liu et al., 2014). There is a wide variation in SFTS case fatality rates, ranging from 5 to 30% depending on the study region, and older patients are more likely to suffer from severe symptoms (Bopp et al., 2020). The WHO has called for urgent research and development of efficient treatments for SFTS due to the lack of specific and effective treatments that have been developed to date. A major challenge is recognizing and treating critically ill patients and those who may die from SFTS. Even though there are already some studies describing the characteristics, predictors, or treatments of SFTS, most of them only discussed the association between fatal outcomes and various indicators, such as viral load, gender, comorbidities, age, and immune predictors that need special detection (He et al., 2020; Hu et al., 2021b; Zhao et al., 2021). In addition, several studies tried to establish a special score model to forecast poor prognosis, which seemed difficult to count and limited in generalization (Wang et al., 2019). It is necessary to find some convenient, effective, and economic indicators to warn of the fatal outcomes of SFTS. The intensive monitoring of these laboratory parameters could help in recognizing the fatal outcome in the early stages. Our retrospective cohort study here focused on determining the predictive value of laboratory indicators in predicting fatal outcomes in patients with SFTS. We aimed to confirm some convenient early-warning biomarkers to help healthcare workers focus on patients with high risks of fatal outcomes more efficiently.

## Methods and Materials

### Patients and Criteria

The retrospective study was performed in two medical centers distributed in Anhui Province: The First Affiliated Hospital of Anhui Medical University and The Second Affiliated Hospital of Anhui Medical University, which are thought to be the two best hospitals in Anhui Province. Patients with confirmed SFTS admitted to the hospital from 1 May 2016 to 31 October 2019 were enrolled in the study. The diagnosis of SFTS was confirmed by: (1) acute fever (temperature >37.5°C for over 24 h) with thrombocytopenia (platelet count <100 × 10^9^/L); and (2) laboratory-confirmed SFTSV infection by the detection of viral RNA, and/or virus-specific IgM antibody in the peripheral blood. The exclusion criteria were: (1) laboratory-confirmed other pathogen infections, such as Platts and Mori rickettsia, Orientia tsutsugamushi, and Hantavirus; (2) human granulocytic anaplasmosis; (3) history of acute or chronic blood system diseases; (4) autoimmune diseases; or (5) other chronic diseases. The studies involving human participants were reviewed and approved by the local Ethics Committee of Anhui Medical University. Informed consent was obtained from all patients following the principles of the Declaration of Helsinki.

### Clinical Data and Laboratory Tests

An examination of medical records was conducted to collect information. The baseline information includes age, gender, residence, and epidemiologic. The clinical symptoms include fever, bellyache, diarrhea, and vomiting. The blood samples were collected after patient admission. The laboratory tests include white blood cell count (WBC), neutrophil count, lymphocyte count, neutrophil to lymphocyte ratio (NLR), platelet count, hemoglobin, aspartate transaminase (AST), alanine aminotransferase (ALT), AST/ALT, lactate dehydrogenase (LDH), γ-glutamyl transpeptidase (GGT), alkaline phosphatase (ALP), albumin (ALB), creatinine kinase (CK), creatinine kinase myocardial b fraction (CK-MB), amylase (AMY), lipase (LIP), blood urea nitrogen (BUN), serum creatinine (Scr), and serum SFTSV viral load. All data were entered by trained study staff. Patients who discontinued therapy or were discharged from the hospital for personal reasons were followed up until 28 days from the start of admission, and death was defined as death from any cause.

### Statistical Analysis

Continuous and categorical variables were presented as median (IQR) and *n* (%), respectively. Categorical variables were compared with the χ^2^ test and continuous variables were compared with the Mann–Whitney *U*-test between survivors and defunct. To explore the risk factors for mortality in patients with SFTS, the indicators were analyzed using univariable and multivariable logistic regression models. Hazard ratios (*HR*s) with 95% *CI*s were calculated using the Cox proportional hazards model. Indicators having *p* < 0.05 in the univariate analysis were included in a multivariate stepwise logistic regression analysis. The predictive values of the potential predictors were evaluated by the receiver operating characteristic (ROC) curve. Differences between the area under the receiver operating characteristic (ROC) curves (AUCs) of the above predictors were tested using the *z*-test. The probability cut-off points for the optimal combination of sensitivity and specificity were determined by the Youden index. The predictive model was validated by the standard diagnostic analysis of sensitivity, specificity, and positive and negative likelihood ratios. Then, Kaplan–Meier survival analysis was used to compare the cumulative risk for mortality both in NLR and viral load, and the significance of the difference was tested with the log-rank test. A *p*-value of <0.05 was considered statistically significant. Statistical analysis was performed using SPSS (version 26.0), PRISM (version 8), and MedClac (version 19.0) software.

## Results

### Characterization of Patients With SFTS

From 1 May 2016 to 31 October 2019, a total of 228 patients with SFTS were diagnosed in the First Affiliated Hospital of Anhui Medical University and Second Affiliated Hospital of Anhui Medical University. All the 228 patients came from the Anhui Province, 143 patients had accepted PCR for testing the SFTSV viral load, and 85 patients had received IgM. A total of 51 patients were enrolled in the death group, 27 died during hospitalization, and 24 died at home after asking for discharge. The mortality rate was 22.37%. The median age of all 228 patients with SFTS was 63 years (IQR 54–71), and the age in the death group (67 years) was numerically higher than the survival group (61 years). The survival group included 82 (46.33%) men and 95 (53.67%) women, respectively, and the death group included 25 (49.02%) men and 26 (50.98%) women. The most common symptom upon admission was fever. All patients with SFTS suffer from fever, whether in the survival group or death group. In addition, digestive system symptoms were the most common clinical manifestations of patients with SFTS, and other symptoms, such as dizziness and myalgia had been confused when patients were suffering from fever. In this article, only bellyache, diarrhea, and vomiting were observed. Diarrhea was the most frequent gastrointestinal symptom, which occurred in 60 (34%) survival patients, and 22 (43%) fatal patients. Bellyache and diarrhea were more common in the death group, in which 9(18%) and 11(22%) fatal patients suffered. The incidence of vomiting was the same between the two groups (Table 1).

**Table 1:** Baseline characteristics of patients with severe fever with thrombocytopenia syndrome (SFTS).

**Characteristics**	**All patients (*n* = 228)**	**Survival** **(*n* = 177)**	**Death (*n* = 51)**	*P* value*
Age, years	63.0 (54.0–70.8)	61.0 (53.0–70.0)	67.0 (61.0–72.0)	0.063
Gender				0.734
Male	107 (47%)	82 (46%)	25 (49%)	
Female	121 (53%)	95 (54%)	26 (51%)	
**Symptoms**				
Fever	228 (100%)	177 (100%)	51 (100%)	1.000
Bellyache	32 (14%)	23 (13%)	9 (18%)	0.399
Diarrhea	82 (36%)	60 (34%)	22 (43%)	0.226
Vomiting	54 (24%)	43 (24%)	11 (22%)	0.687
**Laboratory indicators (normal range)**				
WBC (3.5–9.5 × 10^9^/L)	2.1 (1.4–3.7)	2.1 (1.4–3.8)	1.9 (1.3–3.0)	0.212
Neutrophil count (1.8–6.3 × 10^9^/L)	1.2 (0.8–2.1)	1.1 (0.6–2.0)	1.6 (1.0–2.6)	0.002
Lymphocyte count (1.1–3.2 × 10^9^/L)	0.7 (0.4–1.2)	0.7 (0.4–1.3)	0.5 (0.4–0.8)	0.008
NLR	1.9 (1.2–2.7)	1.6 (1.0–2.3)	2.7 (2.3–3.4)	0.000
PLT (125–350 × 10^9^/L)	44.0 (31.0–54.0)	46.0 (34.0–57.0)	32.0 (23.0–48.0)	0.000
HGB (130–175 g/L)	130.0 (117.0–140.0)	130.0 (117.0–141.0)	128.5 (115.0–136.8)	0.339
AST (13–35 U/L)	186.0 (109.5–372.0)	163.0 (89.0–310.5)	372.0 (169.0–687.0)	0.000
ALT (7–40 U/L)	82.0 (52.0–155.0)	77.0 (49.5–126.5)	118.0 (64.0–223.0)	0.012
AST/ALT	2.3 (1.8–2.9)	2.15 (1.61–2.72)	2.80 (2.12–3.76)	0.000
LDH (120–250 U/L)	912.0 (585.5–1934.0)	820.0 (518.5–1700.0)	1443.0 (862.0–2362.0)	0.000
GGT (7–45 U/L)	32.0 (20.0–62.0)	32.0 (20.0–66.5)	33.0 (22.0–63.0)	0.494
ALP (50–135 U/L)	64.5 (51.0–87.0)	63.0 (50.0–84.0)	71.0 (56.0–93.0)	0.047
ALB (40–55 g/L)	31.6 (28.8–35.6)	32.3 (29.5–36.0)	31.0 (27.5–33.4)	0.037
CK (18–198 U/L)	446.0 (233.0–974.0)	433.0 (233.0–882.5)	555.0 (248.0–1127.0)	0.235
CK-MB (0–25 U/L)	22.0 (11.0–37.0)	22.0 (11.0–37.0)	24.0 (13.0–37.0)	0.516
Amylase (0–150 U/L)	112.0 (75.3–178.0)	111.0 (75.0–178.0)	112.0 (78.0–186.0)	0.818
Lipase (0–1500 U/L)	436.0 (179.0–758.8)	399.0 (173.0–758.5)	519.0 (264.0–814.0)	0.253
BUN (2.6–7.5 mmol/L)	5.8 (4.5–7.6)	5.4 (4.3–7.0)	7.0 (5.4–11.0)	0.000
Scr (41–73 μmol/L)	74.0 (62.4–96.5)	72.0 (61.1–92.0)	84.9 (68.0–136.0)	0.001

Data are n (%) or median (interquartile range, IQR). The group was divided according to survival or death. *p value describes the comparison between survival and death groups. NLR, neutrophil-to-lymphocyte, U/L, units per L.

Compared with the normal range, all patients with SFTS showed lower WBC, neutrophil count, lymphocyte count, and PLT, but higher AST, ALT, LDH, and CK. In addition, the comparison of laboratory tests between the survival group and the death group showed that the level of WBC, lymphocyte count, and PLT dropped more obviously in the death group, and on contrary, the level of neutrophil count and neutrophil-to-lymphocyte ratio (NLR) was higher in the death group. In addition, the level of AST, ALT, AST/ALT, and LDH raised higher in the death group. For other laboratory indicators (ALP, ALB, and BUN), although the median data of all patients with SFTS were in the normal range, ALP and BUN were higher in the death group and ALB was lower in the death group.

### Prognostic Factors for Fatal Outcome in Patients With SFTS

As shown in Table 2, the fatal outcome of patients with SFTS was significantly associated with laboratory indicators, such as NLR, AST, AST/ALT, LDH, BUN, Scr, neutrophil count, lymphocyte count, and platelet count in univariable Cox regression analyses. Furthermore, multivariable Cox regression analysis was used to confirm the relationship between fatal outcomes and laboratory tests. The results indicated that NLR, AST, AST/ALT, and BUN were the independent risk factors for fatal outcomes in patients with SFTS.

**Table 2:** Univariable and multivariable Cox regression analyses of indicators associated with the fatal outcomes of patients with SFTS.

**Laboratory indicators**	**Univariable analysis**		**Multivariable analysis**	
	**HR (95% CI)**	***P* value**	**HR (95% CI)**	***P* value**
Age	1.02 (0.99–1.05)	0.085		
Gender	1.11 (0.64–1.91)	0.722		
Neutrophil count	1.25 (1.06–1.48)	0.009		
Lymphocyte count	0.45 (0.25–0.84)	0.011		
NLR	1.49 (1.32–1.69)	0.000	1.52 (1.32–1.75)	0.000
PLT	0.97 (0.96–0.99)	0.001		
AST	1.00 (1.00–1.00)	0.000	1.00 (1.00–1.00)	0.029
ALT	1.00 (1.00–1.00)	0.092		
AST/ALT	1.43 (1.24–1.64)	0.000	1.34 (1.13–1.60)	0.001
LDH	1.00 (1.00–1.00)	0.000		
ALP	1.00 (0.99–1.01)	0.272		
ALB	0.96 (0.91–1.01)	0.076		
BUN	1.12 (1.06–1.19)	0.000	1.11 (1.05–1.18)	0.001
Scr	1.01 (1.01–1.01)	0.000		

*HR, hazard ratio; NLR, neutrophil-to-lymphocyte*.

**Figure 1:**
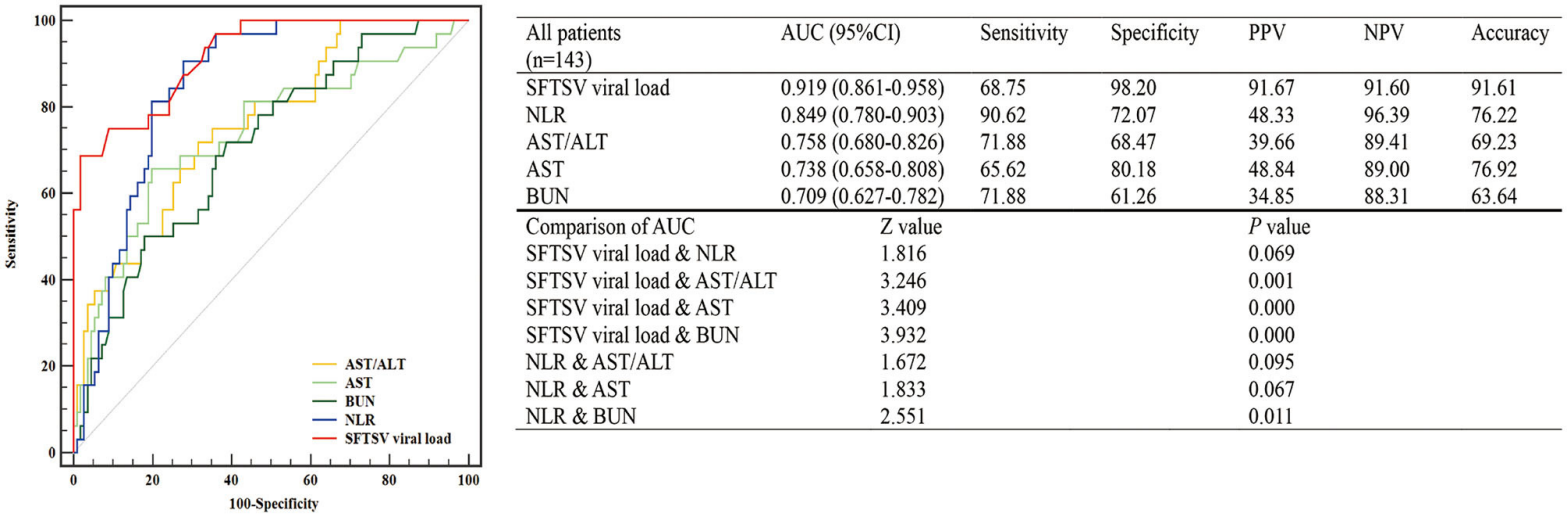
Predictive accuracy of the laboratory indicators of patients with severe fever with thrombocytopenia syndrome (SFTS). The efficacy of laboratory indicators (SFTS virus (SFTSV) viral load, NLR, AST, AST/ALT, and BUN) in predicting the fatal outcome of patients with SFTS was calculated by receiver operating characteristic (ROC) curves. NLR, neutrophil-to-lymphocyte; AST/ALT, aspartate transaminase/alanine aminotransferase; AST, aspartate transaminase; BUN, blood urea nitrogen.

**Figure 2:**
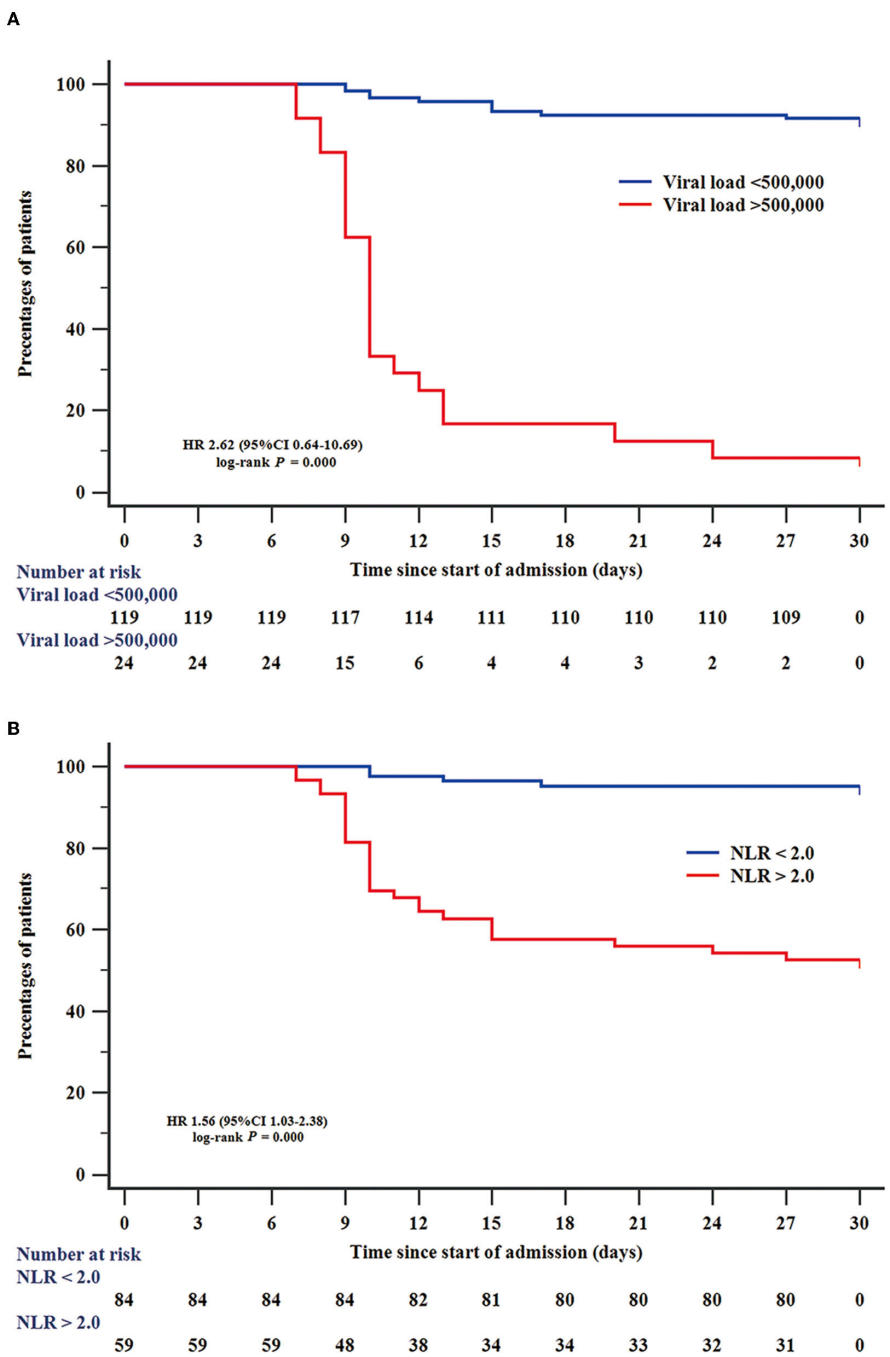
Efficiency of SFTSV viral load and NLR in predicting survival of patients with SFTS. Kaplan–Meier curves were used to analyze the effect of SFTSV viral load **(A)** and NLR **(B)** on predicting survival. NLR, neutrophil-to-lymphocyte.

Among all 228 patients with SFTS, 143 patients with SFTS (111 survival and 32 death) were accepted tests for the SFTSV viral load. To assess the value of laboratory indicators to predict the fatal outcome of patients with SFTS, the SFTSV viral load and indicators which had been confirmed to be associated with fatal outcomes in multivariable Cox regression (NLR, AST, AST/ALT, and BUN) were included in the ROC analysis. As shown in Figure 1, the SFTSV viral load and NLR showed excellent predictive value of the fatal outcome. The AUCs of SFTSV viral load were the highest (0.919), then NLR (0.849), followed by AST/ALT (0.758), AST (0.738), and BUN (0.709). The AUC of viral load was significantly higher than those of AST/ALT, AST, and BUN (*p* < 0.001), and the AUC of NLR was significantly higher than BUN and numerically higher than those of AST/ALT, and AST. The difference in AUC between SFTSV viral load and NLR was not significant. The cut-off values of SFTSV viral load and NLR to predict the fatal outcome were 500,000 and 2.0, respectively. As shown in Figure 2, Kaplan–Meier survival curves were used to confirm that the case fatality rate was significantly increased in patients whose SFTSV viral load was higher than 500,000. Furthermore, lower NLR (<2.0) was significantly associated with improved survival in patients with SFTS.

### Differences in Treatment Between Survival and Death Group

Among 228 patients with SFTS, 177 (78%) patients had used ribavirin, and 188 (82%) patients had used gamma-globulin. The results showed that there was no difference in using the ribavirin between the survival group and the death group (χ^2^ = 1.18, *p* = 0.278). However, accepted treatment by gamma-globulin had shown a significant difference in the two groups (χ^2^ = 4.46, *p* = 0.035). To verify this, we selected ribavirin, gamma-globulin, and a combination of ribavirin and gamma-globulin in multivariable Cox regression analyses, and it confirmed that using gamma-globulin was an independent risk factor for the fatal outcome (*HR* 1.88 [95% *CI* 1.01–3.47]). The cut-off value of the days of using gamma-globulin was 3 days. To stand up to this point, all 228 patients with SFTS were divided into two groups according to the condition that accepted gamma-globulin in the last 3 days. The results showed that mortality was 39% (32/82) in those using this drug for <3 days, but the mortality was 13% (19/146) in those using it for more than 3 days. The mortality was dropped and the difference was statistically significant (χ^2^ = 20.46, *p* = 0.000). Notably, the efficacy of gamma-globulin in the treatment of patients with SFTS needs further studies.

## Discussion

This retrospective cohort study identified several prognostic factors for the fatal outcome in patients with SFTS, especially that NLR was one of the predictors of mortality, which had never been confirmed before. Furthermore, the SFTSV viral load has also been confirmed to be closely associated with the fatal outcome of patients with SFTS. Based on the above results, we suggest that the detection of SFTSV viral load should be considered when the patients suspect the diagnosis of SFTS. However, most patients with SFTS were from rural areas where the tick-borne was epidemic. Considering the detection of SFTSV viral load is costly and time-consuming, which could not be popularized in rural hospitals. NLR, a convenient and direct early-warning biomarker, which could be easily calculated from blood routine examination, should be considered the best choice to predict the risk of the fatal outcome when SFTSV viral load cannot be determined immediately.

In this study, the mortality of patients with SFTS (22.37%) was higher than the case-fatality rate (10~15%) reported by the Zhejiang Provincial Center for Disease Control and Prevention in Zhejiang Province (Sun et al., 2018). We analyzed the possible reason for the divergence because some patients with SFTS chose to discharge and go home when they were facing death. Therefore, this number of fatalities could not be counted by the CDC, and a similar reason had already been mentioned in the previous article by Li et al. (2018). However, it reminds us that it is needed to establish the baseline of case-fatality in Anhui Province based on an observation that included larger sample data. It is worth mentioning that, because most rural hospitals were unable to detect SFTSV and could not make an accurate diagnosis as soon as possible, it is urgently needed for us to discover some convenient and direct predictors of the fatal outcome.

In the present study, only fever, bellyache, diarrhea, and vomiting, which were easy to observe and describe were included in the clinical symptoms. Fever was the most common symptom that patients with SFTS complained about, and all those symptoms were not observed with a significant discrepancy between survival and death groups. Previous studies had also reported similar conclusions (Miao et al., 2021). The NLR is a simple indicator to easily assess the inflammatory status. It has proven its ability in cardiovascular diseases, several cancers, and some infectious pathologies, such as pediatric appendicitis and infective endocarditis. NLR was identified as the independent risk factor for severe illness in patients with COVID-19 in a recent study, and previous studies had illustrated that the increase in NLR indicated poor clinical prognosis (Yang et al., 2020). In this study, we first found that NLR was higher in the death group of patients with SFTS. Interestingly, Liu et al. reported that neutrophil extracellular traps showed a significant association with the poor prognosis in patients with SFTS (Zhang et al., 2020). Our previous studies also found that the inflammation mediators, such as interleukin (IL)-6, IL-10, IL-8, and tumor necrosis factor-α (TNF-α), which are released from immune cells, were increased in patients with SFTS (Hu et al., 2018, 2021a). Combined with the results of the current study, all the pieces of evidence indicate that the role of neutrophils in the progression of SFTS needs further studies.

In the current study, five laboratory indicators (SFTSV viral load, NLR, AST/ALT, AST, and BUN) were enrolled to compare the efficacy in predicting the fatal outcome of patients with SFTS. The results showed that SFTSV viral load and NLR had higher AUC, sensitivity, and specificity, which indicated that SFTSV viral load and NLR were better than the other three indicators in predicting the incidence of fatal outcomes. The importance of the detection of SFTSV viral load had been repeatedly mentioned in previous studies (Huang et al., 2022). It is worth mentioning that only 143 patients among the enrolled 228 patients with SFTS had accepted SFTS viral load test in this study. SFTS is an emerging infectious disease which had been reported to be mainly transmitted by contact with ticks. Following the characteristics of this infection, a considerable number of SFTS patients are farmers who are exposed to ticks during work in the field. Some of them declined the test because of its high price. The possibility of this selection bias had also been considered. As shown in Supplementary Table 1, some baseline characteristics and NLR were compared between patients who accepted SFTS viral load test and those who did not, and the results showed that there was no difference between the two groups.

Additionally, the PCR test for SFTS viral load is not available in rural hospitals. Interestingly, this is the first time that NLR is deemed as a convenient early-warning biomarker for patients with SFTS since it is a much cheaper test and can also be conducted in rural hospitals. Based on the results, we suggest that patients with SFTS can improve risk stratification and management according to NLR and SFTSV viral load. Patients with SFTSV viral load <50,000 or NLR <2.0 are less likely to be at risk of fatal outcomes. Patients with SFTSV viral load ≥ 50,000 and NLR ≥ 2.0 should be actively offered more attention and support equipment since they are at a higher risk of fatal outcomes. If there are large-scale cases, the risk stratification and management will help alleviate the shortage of medical resources and reduce the mortality of critical patients. Further, in several rural hospitals, which are unable to measure the SFTSV viral load, NLR testing will be an effective means of predicting the fatal outcome of SFTS.

The effectiveness of gamma-globulin in treating SFTS has not been reported, despite its extensive use as a clinical treatment in China. In this study, the efficacy of gamma-globulin in the treatment of patients with SFTS had been observed. It is concurrently confirmed that the effect of ribavirin had no difference between the survival and death groups. The duration of gamma-globulin that had been proposed should not be <3 days. More importantly, the clinical improvement of gamma-globulin in patients with SFTS requires confirmation in larger studies.

In summary, our work first provided evidence that NLR is a convenient early warning biomarker for the fatal outcome of patients with SFTS. Using NLR as a predictor has the following advantages: (1) the detection of NLR is very easy and not expensive and can be extended to rural hospitals, (2) the interpretation of NLR results is simple without complex calculation, and (3) it could be used as a preliminary judgment of the prognosis of patients with SFTS, which help doctor focus on patients with a high risk of a fatal outcome more efficiently. Furthermore, we revealed the important role of gamma-globulin in the treatment of SFTS, which had been previously ignored, which could bring new hope for specific treatment for patients with SFTS to reduce the high mortality.

## Data Availability Statement

The original contributions presented in the study are included in the article/Supplementary Material, further inquiries can be directed to the corresponding authors.

## Ethics Statement

The studies involving human participants were reviewed and approved by the local Ethics Committee of Anhui Medical University. The patients/participants provided their written informed consent to participate in this study.

## Author Contributions

JL and YG contributed to the study concept and design. ZW, LK, LHe, NS, and SM contributed to the acquisition of clinical data, and YW and JQ contributed to statistical analysis and data interpretation. YW and ZW wrote the first draft of the manuscript. HX, LHu, GZ, YG, and JL supervised and oversaw the study. All authors contributed to manuscript revision, and read and approved the submitted version.

## Funding

This study was supported by the National Natural Science Foundation of China (Youth Program) (81900554), the Major Program for Supporting Outstanding Talents in Colleges of the Ministry of Human Resources and Social Security of the People's Republic of Anhui (gxyqZD2020013), and the Central Guidance on Local Science and Technology Development Fund (9021039203).

## Conflict of Interest

The authors declare that the research was conducted in the absence of any commercial or financial relationships that could be construed as a potential conflict of interest.

## Publisher's Note

All claims expressed in this article are solely those of the authors and do not necessarily represent those of their affiliated organizations, or those of the publisher, the editors and the reviewers. Any product that may be evaluated in this article, or claim that may be made by its manufacturer, is not guaranteed or endorsed by the publisher.
