# Corrigendum: NLR, a convenient early-warning biomarker of fatal outcome in patients with severe fever with thrombocytopenia syndrome

**DOI:** 10.3389/fmicb.2025.1553518

**Published:** 2025-01-27

**Authors:** Yuanyuan Wei, Zilong Wang, Luyang Kang, Lingling He, Nan Sheng, Jiangfeng Qin, Shuangshuang Ma, Honghai Xu, Lifen Hu, Guizhou Zou, Yufeng Gao, Jiabin Li

**Affiliations:** ^1^Department of Hospital Infection Prevention and Control, The First Affiliated Hospital of Anhui Medical University, Hefei, China; ^2^Anhui Center for Surveillance of Bacterial Resistance, The First Affiliated Hospital of Anhui Medical University, Hefei, China; ^3^Department of Infectious Diseases, The First Affiliated Hospital of Anhui Medical University, Hefei, China; ^4^Department of Infectious Diseases, The Second Affiliated Hospital of Anhui Medical University, Hefei, China; ^5^Department of Pathology, The First Affiliated Hospital of Anhui Medical University, Hefei, China

**Keywords:** severe fever with thrombocytopenia syndrome, neutrophil-to-lymphocyte ratio, SFTSV viral load, prognosis factors, fatal outcome

In the published article, there was an error in affiliation [2]. Instead of “[Anhui Center for Surveillance of Bacterial Resistance, Hefei, China]”, it should be “[Anhui Center for Surveillance of Bacterial Resistance, The First Affiliated Hospital of Anhui Medical University, Hefei, China]”.

The authors apologize for this error and state that this does not change the scientific conclusions of the article in any way. The original article has been updated.

