# Supplementary material for: NLR, A Convenient Early-Warning Biomarker of Fatal Outcome in Patients With Severe Fever With Thrombocytopenia Syndrome
Source: Front Microbiol. 2022 Jun 23;13:907888. doi: 10.3389/fmicb.2022.907888 (PMC9262381; doi:10.3389/fmicb.2022.907888)
Supplement: Supplementary file 1 [file Table_1.docx]

| **Characteristics** | **Accepted SFTSV viral load test**  **(n=143)** | **Did not accept SFTSV viral load test**  **(n=85)** | ***P***  **value^*^** |
| --- | --- | --- | --- |
| Age, years | 63.0 (54.0-71.0) | 62.0 (53.5-70.0) | 0.562 |
| Death patients | 32 (22%) | 19 (22%) | 0.997 |
| Gender |  |  | 0.259 |
| Male | 63 (44%) | 44 (52%) |  |
| Female | 80 (56%) | 41 (48%) |  |
| NLR | 1.8 (1.1-2.7) | 2.0 (1.4-2.6) | 0.260 |

Supplement table1. The comparison between patients who accepted SFTSV viral load test and those who did not.

Data are n (%) or median (IQR). Group was divided according to patients who accepted SFTSV viral load test and those who did not. * *P* value describes the comparison between those two groups. NLR: neutrophil-to-lymphocyte.
